# Feasibility of Interleaving Computerized Cognitive Training With Repetitive Transcranial Magnetic Stimulation: Pilot Studies in Mild Cognitive Impairment Due to Alzheimer’s Disease and Stroke

**DOI:** 10.2196/81437

**Published:** 2026-07-15

**Authors:** Laura M Campbell, Stephanie Aghamoosa, Olivia Horn, Holly H Fleischmann, James Lopez, Katrina S Rbeiz, Katrina Madden, Michael Antonucci, Gonzalo Revuelta, Lisa M McTeague, Andreana Benitez

**Affiliations:** 1 Department of Psychiatry, University of California, San Diego, La Jolla, CA, United States; 2 Department of Psychiatry and Behavioral Sciences, Medical University of South Carolina, Charleston, SC, United States; 3 Department of Health Sciences and Research, Medical University of South Carolina, Charleston, SC, United States; 4 Department of Neurology, Medical University of South Carolina, Charleston, SC, United States; 5 Department of Psychology, University of Georgia, Athens, Georgia, United States; 6 Department of Radiology and Radiological Science, Medical University of South Carolina, 96 Jonathan Lucas St, Charleston, SC, 29425, United States, (843) 876-8479

**Keywords:** cognitive remediation, cognitive training, mild cognitive impairment, Alzheimer’s disease, stroke, neuromodulation, transcranial magnetic stimulation, mHealth, feasibility studies, older adults

## Abstract

**Background:**

Repetitive transcranial magnetic stimulation induces neural plasticity, which may be maximized via adjunctive interventions.

**Objective:**

This study aimed to examine the feasibility and acceptability of interleaving computerized cognitive training (CCT) with accelerated intermittent theta burst (iTBS) repetitive transcranial magnetic stimulation in 2 open-label phase I trials of amnestic mild cognitive impairment (aMCI; NCT04503096) and poststroke mild cognitive impairment (psMCI; NCT04655963).

**Methods:**

Participants (aMCI: n=21; and psMCI: n=14) received 24 sessions of accelerated iTBS over 3 days (8 sessions per day). During the 10‐ to 15-minute intervals between iTBS sessions, participants engaged in CCT via the BrainHQ platform. During the 4-week follow-up period, participants were asked to complete 20 minutes daily of BrainHQ. Both studies offered identical compensation for daily practice, but the aMCI group received weekly reminders, whereas the psMCI group did not. Study-specific questionnaires assessed BrainHQ acceptability (administered post-treatment and at follow-up).

**Results:**

During in-laboratory treatment, the groups completed similar amounts of BrainHQ (aMCI: median 107, IQR 92-121 min; and psMCI: median 111, IQR 93-137 min; *P*=.51). However, engagement differed during the unmonitored follow-up (aMCI: median 450, IQR 167-581 min,; and psMCI: median 142, IQR 0-477 min); practice time increased in the aMCI group but decreased in the psMCI group over the 4 weeks (*P*=.001). Both groups generally rated BrainHQ positively at both time points in terms of their experience with the platform, exercises, and perceived benefit for their mood and functioning.

**Conclusions:**

Combined treatment with CCT and accelerated iTBS is feasible and acceptable in people with mild cognitive impairment. Standardized dosing of CCT is feasibly achieved when interleaved with iTBS sessions and completed in-laboratory, but ecological adherence to CCT in an unmonitored, self-directed context likely requires additional supports and may be influenced by patient characteristics.

## Introduction

Repetitive transcranial magnetic stimulation (rTMS) of the left dorsolateral prefrontal cortex (l-dlPFC) is a non-invasive brain stimulation technique that can induce neuroplasticity [1]. While rTMS has primarily been studied within psychiatric disorders (eg, treatment-refractory depression), there is growing interest in its use as a treatment for cognitive impairments in neurological disorders, such as mild cognitive impairment (MCI) due to Alzheimer’s disease (AD) [2] and stroke [3,4]. The evidence base in these neurological conditions is nascent but steadily growing. Early meta-analyses of sham-controlled trials of rTMS to enhance cognitive functions in these conditions have found medium-to-large effect sizes for MCI and AD dementia [2,4,5] and more modest but nonetheless positive effects in stroke [3,4,6,7]. This is particularly promising, given that many other therapies have limited efficacy in these populations [8-10].

While these results are encouraging, there remains a significant need to optimize the efficacy of rTMS to l-dlPFC for cognitive impairment. One approach for doing so is to incorporate an adjunctive treatment to enhance its effects. As rTMS can induce neuroplasticity in targeted cognitive circuitry [11], incorporating computerized cognitive training (CCT) as an adjunctive intervention may work synergistically to enhance cognitive gains. CCT uses a restorative approach to cognitive remediation through repeated practice of tasks or exercises in specific cognitive domains using a technological device (eg, a computer or tablet) [12].

CCT has been studied within the context of cognitive impairment due to AD and stroke. With adequate training and optimization of the CCT to fit the needs of the intended population, CCT in middle-aged and older adults with and without MCI is feasible [13-15]. However, the efficacy of these interventions for MCI is less clear, as some studies report improvements in cognition, albeit with small effect sizes, whereas other studies do not [16-20]. Likely sources of this variability in findings are the heterogeneity of research design features, such as CCT platform, exercises, and dose. Notably, a recent meta-analysis of randomized or quasi-randomized trials with healthy older adults demonstrated that 2 platforms, BrainHQ and CogniFit—in contrast to other commercially available options—have the highest quality evidence supporting their efficacy [21].

CCT that trains specific cognitive domains is presumed to enhance performance on similar tasks via neuroplasticity [16]. As rTMS has also been shown to induce neuroplasticity [22], it is therefore plausible that combining CCT with rTMS may yield greater cognitive gains than either rTMS or CCT alone. CCT is a particularly attractive complement to rTMS, given that it poses minimal clinical burden, is cost-effective and low risk, and can be self-administered in between rTMS sessions. However, to date, CCT and rTMS have mostly been studied in isolation, and there is only limited literature examining the combination of these interventions [23]. Moreover, the majority of studies have focused on once-daily rTMS over weeks, which can be quite burdensome, as it requires daily trips to a laboratory or clinic for treatment. Newer rTMS protocols, such as accelerated intermittent theta burst stimulation (iTBS), which delivers similar or higher doses than conventional rTMS in as little as 3 multi-session days [24], are better suited for combination with CCT, as it can be interleaved between same-day iTBS sessions. Critically, the feasibility and acceptability of combined accelerated iTBS and CCT have yet to be established, which is an important first step. For example, the common, albeit mild and transient, side effects of rTMS (eg, headache [25]) may impact engagement with CCT; therefore, it should not be assumed that the feasibility and acceptability of each intervention in isolation apply to their joint delivery.

To this end, we conducted 2 open-label phase I clinical trials of accelerated iTBS to the l-dlPFC with interleaved CCT via the empirically supported BrainHQ platform in adults with amnestic MCI (aMCI) due to possible AD (NCT04503096) or poststroke MCI (psMCI; NCT04655963). While the primary aim of these studies, with parallel designs, was to establish the safety of accelerated iTBS treatment in these groups, an exploratory aim was to examine the feasibility and acceptability of interleaved CCT. Accelerated iTBS was delivered with multiple same-day sessions separated by 10‐ to 15-minute intersession intervals, making it ideally suited for combination with CCT. As such, we administered BrainHQ in a standard, supervised manner during the intervals between 8 accelerated iTBS sessions on each of 3 treatment days. At post-treatment, participants were asked to independently engage in BrainHQ for 20 minutes daily over a 4-week follow-up period. The purpose of including this follow-up phase was to assess ecological adherence to CCT outside of the monitored laboratory setting. This can inform the design of future trials that are likely to include CCT engagement outside of iTBS treatment visits, capitalizing on the post-iTBS window of neuroplasticity and enabling an increase in CCT dose to potentially efficacious ranges.

Here, we report data on feasibility (ie, retention and adherence), engagement (ie, usage patterns and types of programs and exercises completed), and acceptability ratings observed over the treatment phase and the 4-week follow-up period. Due to the standardized administration of BrainHQ during accelerated iTBS treatment, a post-treatment compensation schedule that incentivized use, modest daily use expectations, and a comparable degree of cognitive function between groups (ie, no dementia), we hypothesized that engagement in BrainHQ during and post-treatment would be comparable across both MCI groups. We similarly expected that interleaved BrainHQ would be rated as acceptable on questionnaires that assess participants’ experiences.

## Methods

### Ethical Considerations

Both studies were approved by the Medical University of South Carolina’s (MUSC) Institutional Review Board and were pre-registered in ClinicalTrials.gov (aMCI: NCT04503096; and psMCI: NCT04655963; IRB Pro00100536 and Pro00083136, respectively). All participants provided written informed consent and were informed of their right to withdraw from the study at any time without penalty. Participant privacy and confidentiality were strictly maintained in accordance with IRB requirements, and no personally identifiable information is reported in this manuscript or supplementary materials. Participants were offered compensation at the rate of US $4.50 per completed 20-minute session per day, equating to up to US $126 total. Efforts to reduce potential sources of bias in these open-label trials included (1) minimizing selection bias by using rigorous and predefined eligibility criteria applied consistently to all participants; (2) minimizing confounding factors by constraining disease severity to MCI and excluding comorbidities likely to impact the study outcomes; and (3) avoiding reporting bias through trial pre-registration and a transparent and complete description of the study procedures, outcome measures, hypotheses, planned analyses, and results. The primary results from the aMCI trial focused on safety, feasibility, and acceptability of iTBS are reported in a study by Aghamoosa et al [26]. The description of the BrainHQ methods adheres to the Template for Intervention Description and Replication (TIDieR) checklist [27], as described below and summarized in Table S1 in Multimedia Appendix 1.

### Participants

#### Overview

Trained coordinators screened potential participants for eligibility over the phone and using the participant’s electronic health record, with appropriate permissions. The following 2 paragraphs describe the eligibility criteria that were unique to each study, and the last paragraph describes the criteria shared by both studies. Target sample sizes were selected to generate estimates of the trials’ primary outcomes (ie, accelerated iTBS treatment feasibility, acceptability, and outcome variability) for use in planning subsequent trials.

#### aMCI Participants

Twenty-two participants enrolled in the study and initiated treatment, and 21 completed all procedures. Participants were recruited from MUSC Neurology and Neuropsychology outpatient clinics. Participants had to be aged between 60 and 85 years and diagnosed with MCI due to possible AD by a neurologist or neuropsychologist within the last 2 years, using National Institute on Aging and Alzheimer's Association (NIA-AA) criteria [28]. They also needed to meet actuarial neuropsychological criteria for aMCI upon clinical assessment, which requires impairment (≤16th percentile using demographically corrected norms) in ≥2 scores within one cognitive domain or ≥1 score in ≥3 domains, one of which being memory [29]. The primary suspected cause had to be possible or probable AD, with other primary, competing etiologies (eg, psychiatric, movement disorder, stroke, and reversible causes) ruled out.

#### psMCI Participants

Twenty-one participants enrolled in the study; 15 (71%) initiated treatment, and 14 (67%) completed all procedures. Participants were recruited from the National Institutes of Health (NIH)–funded COBRE for Stroke Recovery Registry at MUSC and from the community (eg, advertisements, community events, and social media). All participants had to be aged ≥40 years and have a history of ischemic or hemorrhagic stroke (with intact cortex under the transcranial magnetic stimulation coil) with at least 6-month chronicity. Acute or subacute hemorrhage or any age bilateral hemorrhage or hemorrhage with mass effect, confirmed with scanning, was exclusionary. Participants had to retain the ability to perform the cognitive tasks (eg, no moderate to severe global aphasia or visual impairment). Although not required for enrollment, we retrospectively confirmed that all psMCI participants met actuarial neuropsychological criteria for MCI [29] on the neuropsychological testing completed during the study.

For both groups, contraindications to magnetic resonance imaging (MRI) or rTMS were exclusionary, including electrically, magnetically, or mechanically activated metal or nonmetal implants; pregnancy; claustrophobia; history of a seizure disorder; or preexisting scalp lesion, wound, bone defect, or hemicraniectomy. Additional exclusionary criteria included a clinical diagnosis of major neurocognitive disorder or dementia, history of significant or unstable medical conditions that could have affected cognition (eg, lifetime substance or alcohol use disorder, severe mental illness, other neurological disorders, severe cardiometabolic disorders, and developmental disorders), no detectable active or passive motor threshold in the paretic or nonparetic right hand, and current enrollment in a clinical trial that involved any investigational treatment. Participants were excluded if they endorsed daily or weekly use of anticholinergics, neuroleptics, sedative-hypnotics, bupropion, or stimulants (unless stimulant use was deemed low risk by the coinvestigator physician safety monitor). Use of cholinesterase inhibitors, N-methyl-D-aspartate (NMDA) receptor antagonists, and antidepressants was permitted if the participant was on a stable regimen for at least 4 weeks prior to enrollment. Participants were required to have English as their first or primary language.

### Procedures

#### Study Design Overview

These 2 pilot studies were designed to have parallel procedures, although they were not conducted simultaneously and were not identical. There were minor differences in some study procedures (eg, experience questionnaire and delivery of BrainHQ post-treatment) arising from differences in study aims and refinements to the aMCI study based on lessons learned from the psMCI study, which started first. These trials were open-label phase I studies (see a study by Aghamoosa et al [26] for details). Potential participants underwent screening to determine eligibility and provided written informed consent. All in-person procedures were conducted in research-designated space at an academic medical center. Participants completed the testing of resting motor threshold at pretreatment. Pre- and posttreatment assessments (including self-reported demographic characteristics and medical history, neuropsychological testing, neuropsychiatric interviews and questionnaires, and brain MRI) were completed within 1 week of treatment initiation and termination. Participants completed 3 accelerated iTBS treatment days with interleaved CCT. In the interest of feasibility and acceptability, participants chose which 3 days within an 8-day span to receive treatment. For the 4 weeks after treatment, participants were asked to complete BrainHQ for 20 minutes per day at home using their own device and completed weekly online questionnaires. Study iPad Minis were available if participants did not have their own device, but no participants chose this option. At the end of the 4-week monitoring period, they completed a telehealth follow-up assessment (questionnaires and a global cognitive screening measure). Participants were compensated for completion of each study visit and engagement in BrainHQ during the follow-up period (as described in the following sections). The aMCI participants completed the study between 2021 and 2022. All psMCI participants completed the study in 2021.

#### Accelerated iTBS Treatment

Both groups received identical accelerated iTBS treatment. Each participant completed 3 accelerated iTBS treatment days (~2 h per day), each consisting of 8 iTBS sessions with 10‐ to 15-minute inter-session intervals (24 total sessions). Treatment was delivered using a MagVenture MagPro TMS System with a figure-8 coil. We administered 600 pulses per session (14,400 total pulses) in 50 Hz triplets at 120% of resting motor threshold (2-s trains repeated every 10 s with 8-s inter-train intervals for 190 s). Stimulation was targeted to the l-dlPFC at F3 using scalp measurements from the 10 to 20 electroencephalogram (EEG) system [30]. The Brainsight NeuroNavigation System (Rogue Research Inc) was used to ensure precise and consistent coil placement across sessions.

#### CCT via BrainHQ

Participants engaged in Posit Science’s online BrainHQ exercises (version 7.13; Posit Science Corporation). During the in-laboratory treatment phase, BrainHQ was delivered in 10‐ to 15-minute intervals between each of the 8 accelerated iTBS sessions (ie, 7 BrainHQ sessions per treatment day for 210‐315 total minutes) on a study-provided iPad (10.2-inch iPad, 8th generation, iOS 16.4; Apple Inc). After each accelerated iTBS session, study staff handed the iPad to participants to complete the BrainHQ exercises and remained present to answer any questions. During the 4-week follow-up period, participants were asked to independently complete at least 20 minutes of BrainHQ exercises per day (7 sessions per week, 28 total sessions, and 560 total minutes) on their own device (ie, computer, tablet, or smartphone) in their preferred location.

The BrainHQ platform is commercially available and includes exercises in 6 BrainHQ-defined categories: attention, brain speed, memory, people skills, intelligence, and navigation. Most exercises require an upper extremity motor response (eg, touch screen or mouse click), and many require speeded responses even if they are not in the “brain speed” domain; hence, there is some overlap in the cognitive processes being trained across domains. Participants are provided feedback on their performance on each exercise, and the exercises are adaptive, becoming more difficult if performance improves. BrainHQ records the number of seconds engaged in each exercise. A detailed description of each exercise and additional information about the BrainHQ platform can be found on the website provided earlier.

Participants were shown how to use the BrainHQ platform by study staff, who were available to answer questions as needed. Additionally, the BrainHQ platform includes instructions and a sample item for each exercise. During the treatment phase, participants were instructed to use the adaptive “personal trainer” option that selects exercises and difficulty levels based on past performance. During the 4-week follow-up phase, aMCI participants were instructed that they could use either the personal trainer or the “a la carte” option, in which participants select which exercise they would like to complete. psMCI participants were instructed to continue to use the personal trainer during the follow-up phase, but they did have access to the à la carte option. CCT during the follow-up phase was self-administered at the participants’ preferred location and was not actively monitored but was recorded via the BrainHQ platform. In the aMCI study, participants were reminded to complete the BrainHQ activities via an automated weekly email. The psMCI study did not send BrainHQ reminders. BrainHQ was not tailored beyond the built-in adaptivity, and no modifications were made to the BrainHQ protocol during the study.

### Measures

#### Sample Characterization

The NINDS (National Institute of Neurological Disorders and Stroke) and Canadian Stroke Network neuropsychological test battery [31] was used to characterize baseline cognitive function. This battery includes the Hopkins Verbal Learning Test-Revised, Digit Span Forward and Backward, Digit Symbol Coding, Trail Making Tests A & B, semantic (animal) fluency, and phonemic (letter) fluency. Age-adjusted norms provided in the test’s manual were used to correct Hopkins Verbal Learning Test-Revised scores (immediate recall, delayed recall, retention, and recognition), while other scores were corrected for age, sex, and years of education using normative calculators published in the literature (ie, Digit Span and Digit Symbol Coding scores [32] and Trail Making Tests and verbal fluencies [33]). We report normative percentile scores for each test.

#### Feasibility

We assessed the feasibility of combining BrainHQ with accelerated iTBS by evaluating study retention and adherence. Retention was measured as the percentage of participants who initiated treatment and completed all study procedures. Adherence to BrainHQ was assessed separately for each MCI group and for the in-laboratory treatment phase and 4-week follow-up phase, the latter being referred to as ecological adherence. For the treatment phase, adherence was measured as the percentage of prescribed minutes completed out of the total 210 minutes across the 3 treatment days. We could not compute adherence to the number of same-day sessions during the treatment phase as the data were labeled by treatment day, not individual sessions, precluding the enumeration of completed sessions. For the follow-up phase, ecological adherence was measured as the percentage of target minutes completed out of the target total of 560 minutes. We also report the percentage of participants completing all 28 daily sessions and the number of completed sessions (ie, how many days trained) over the 4-week follow-up period.

#### Engagement

To explore how participants interacted with BrainHQ, we evaluated several aspects of engagement across the 2 MCI groups. These included the overall patterns of practice time, the types of exercises completed, and the devices used to complete practice during the treatment and follow-up phases.

#### Acceptability

To evaluate the acceptability of BrainHQ, participants were asked to complete study-specific questionnaires that solicited feedback on their experience. Each questionnaire was administered immediately after the treatment phase and again at the conclusion of the 4-week follow-up period. The questionnaire used in the psMCI study was developed first and then modified for the aMCI study, considering feedback from the initial questionnaire and differences in the type of experience data sought by each study. Both questionnaires inquired about the impact of BrainHQ on functioning, participants’ experiences (such as enjoyment and frustration) with the exercises, and the overall usability experience with the BrainHQ platform.

### Statistical Analyses

Differences in demographic and clinical characteristics between groups were examined using Wilcoxon rank-sum tests and chi-squared or Fisher exact tests, as appropriate. We present descriptive statistics to summarize adherence, engagement, and acceptability for each group. Wilcoxon rank-sum tests were used to compare the total number of minutes of engagement with the BrainHQ exercises between the aMCI and psMCI groups during the supervised treatment phase. During the unsupervised follow-up phase, a rank-based nonparametric alternative to a repeated-measures ANOVA was performed using the *nparLD 2.2* package in R (4.4.2), which produces ANOVA-type statistics (ATS) [34]. This analysis tested whether there was a difference in the number of minutes of engagement in BrainHQ by the MCI group across all weeks as well as whether engagement changed across the weeks by group (ie, a group-by-week interaction).

## Results

### Participants

Demographic and cognitive characteristics are summarized in Table 1. Consistent with the differing age range criteria in the 2 studies, participants in the aMCI group were older than those in the psMCI group. The groups did not differ significantly by sex, and both groups were predominantly non-Hispanic White. Consistent with their distinct underlying etiologies, the 2 MCI groups demonstrated differences in cognitive functioning (ie, poorer memory and semantic fluency in the aMCI group and poorer processing speed in the psMCI group).

**Table 1.** Demographic and cognitive characteristics.

Demographic or cognitive characteristics	aMCI^a^ (n=21)	psMCI^b^ (n=14)	*P* value
Age (y), median (IQR)	72 (69.5‐78.5)	65 (61.3‐72)	.003^c^
Sex n (%)			.16
Male	11 (52%)	11 (79%)
Female	10 (48%)	3 (21%)
Education, median (IQR)	16 (14-17)	14.5 (12-16)	.19
Race or ethnicity, n (%)			.07
Non-Hispanic White	20 (95)	10 (71)	—^d^
Non-Hispanic Black or African American	1 (5)	4 (29)	—
Neuropsychological battery, median percentile (IQR)			
HVLT-R^e^ immediate recall	3 (1-14)	14 (1.75‐32.75)	.08
HVLT-R delayed recall	0 (0‐0)	1 (0.75‐10.5)	.001**^c^**
HVLT-R retention	0 (0‐0)	5 (0‐7)	.003**^c^**
HVLT-R recognition	0 (0‐3.5)	6.5 (0‐15)	.07
Digits forward	22 (8-37)	39.5 (12.75‐53)	.13
Digits backward	24 (14‐43.5)	19.5 (7‐47.75)	.39
Trails A	12 (0‐56)	2.5 (0‐6.25)	.10
Trails B	2 (0‐42.5)	10 (0‐28.25)	≥.99
Digit symbol coding	31 (11-41)	3.5 (1‐22.25)	.03^c^
Animal fluency	8 (2.5‐19.5)	27.5 (12.75‐34.25)	.006^c^
Letter fluency	28 (11.5‐52)	38.5 (12.5‐68.25)	.34

a aMCI: amnestic mild cognitive impairment.

b psMCI: post-stroke mild cognitive impairment.

c *P*<.05.

d Not applicable.

e HVLT-R: Hopkins Verbal Learning Test-Revised.

### BrainHQ Feasibility

Retention was high in both studies. One treatment initiator withdrew from the aMCI group (ie, 21/22, 95.5% completed all study procedures). All participants in the psMCI group who initiated treatment completed study procedures (ie, 14/14, 100%).

Adherence to BrainHQ during the in-laboratory treatment phase was similar in the 2 groups, with both groups completing approximately half of the prescribed 210 minutes: aMCI group median: 51.1% (IQR 43.6%‐57.6%; range 12.9%‐79.5%) of minutes and psMCI group median: 52.9% (IQR 44.4%‐65.3%; range 44.1%‐78.4%; see see Table 2 for the median number of minutes). No participant in either group completed 210 minutes during the treatment phase. Ecological adherence to the target 560 minutes of BrainHQ during the 4-week follow-up phase was higher in the aMCI group (median 80.2%, IQR 29.9%‐103.7% of minutes; range: 0%‐172.4%) than in the psMCI group (median 25.3%, IQR 0%‐85.3% of minutes; range: 0%‐192.4%). The percentage of participants who completed the target 560 minutes over the 4 weeks was 33.3% in the aMCI group and 21.4% in the psMCI group. The percentage of participants who met weekly goals (ie, target 140 min/wk) was variable in the aMCI group: week 1, 2 (10%) participants; week 2, 12 (57%) participants; week 3, 5 (24%) participants; and week 4, 7 (33%) participants. The percentage of psMCI participants who completed the target 140 minutes each week was lower and relatively stable: week 1, 3 (21%) participants; week 2, 5 (36%) participants; week 3, 3 (21%) participants; and week 4, 2 (14%) participants. In both groups, no participant (ie, 0%) completed the target 28 sessions of daily BrainHQ. The aMCI group median number of sessions was 16 (IQR 9‐21), and the median for the psMCI group was 4 (IQR 1‐14).

**Table 2.** Median minutes of BrainHQ during repetitive transcranial magnetic stimulation (rTMS) treatment and in 4-week follow-up.

	aMCI^a^ (n=21)			psMCI^b^ (n=14)		
	Minutes, median (IQR)	Range	Participants who did not do BrainHQ, n (%)	Minutes, median (IQR)	Range	Participants who did not do BrainHQ, n (%)
rTMS treatment phase						
Treatment day 1	25.7 (19.6‐33.9)	6.25‐47.4	1 (5)	42.4 (32.2‐49.7)	22.8‐59.1	2 (14)
Treatment day 2	38.5 (32.4‐45.9)	13.9‐59.6	0 (0)	40.1 (27.6‐51)	17.7‐60.4	1 (7)
Treatment day 3	42.8 (32.2‐52.8)	10‐70.5	0 (0)	40.2 (27.8‐56.3)	19.8‐73	0 (0)
Total	107.2 (91.6–121.0)	27.2-167.0	0 (0)	111.0 (93.2-137.2)	44.1–164.7	0 (0)
Follow-up phase						
Week 1	34.3 (0‐97.6)	0‐178.6	7 (33)	28.6 (0‐109.8)	0‐282.9	5 (36)
Week 2	152.4 (71.3‐177.5)	0‐392.7	3 (14)	27.5 (0‐214.9)	0‐350.8	7 (50)
Week 3	116.4 (49.2‐142.6)	0‐241.9	4 (19)	0 (0‐94.4)	0‐269	8 (57)
Week 4	124.9 (63.2‐163.4)	0‐287.9	2 (10)	0 (0‐56.2)	0‐281.1	9 (64)
Total	449.5 (167.3-580.5)	0-965.4	1 (5)	142.0 (0-477.4)	0-1077.7	5 (36)

a aMCI: amnestic mild cognitive impairment.

b psMCI: post-stroke mild cognitive impairment.

### BrainHQ Engagement

**Figure 1.**
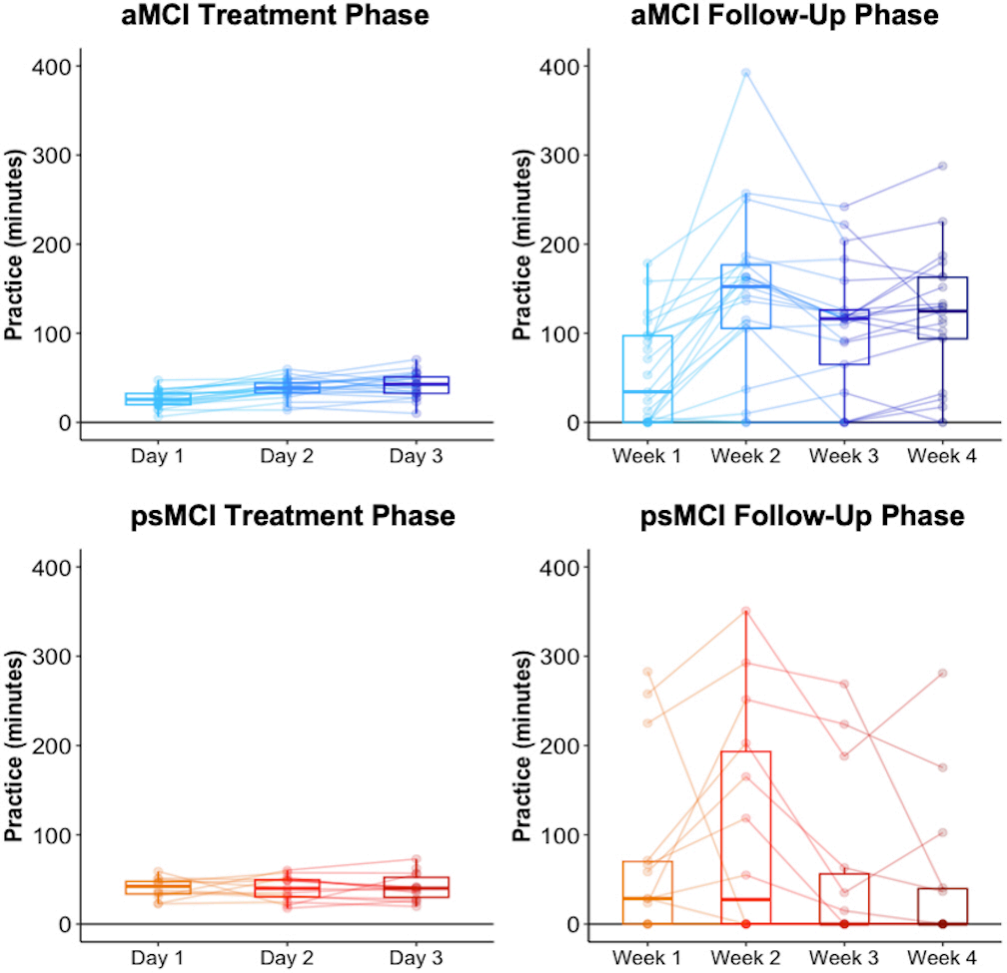
Median (IQR) minutes of BrainHQ engagement during repetitive transcranial magnetic stimulation treatment and in 4-week follow-up. Missing datapoints were excluded from the treatment phase figures. aMCI: amnestic mild cognitive impairment; psMCI: post-stroke mild cognitive impairment.

**Figure 2.**
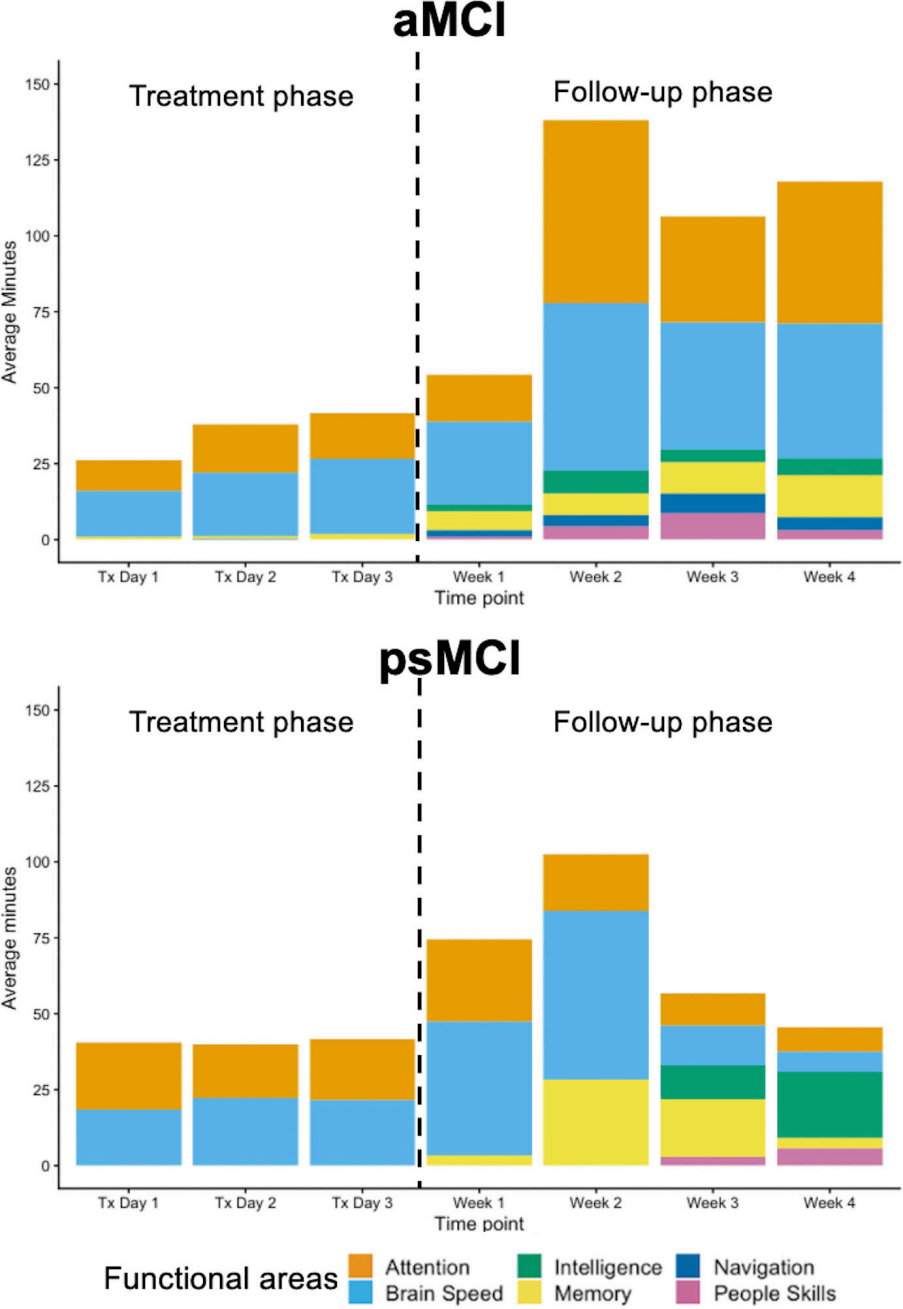
Mean minutes of BrainHQ engagement by exercise domain. aMCI: amnestic mild cognitive impairment; psMCI: post-stroke mild cognitive impairment; Tx: treatment.

The median and IQR minutes of BrainHQ exercises completed on each treatment day and during the 4-week follow-up period are included in Table 2 and depicted in Figure 1. The mean minutes spent engaging in exercise domain on each treatment day and during each week of the 4-week follow-up period are depicted in Figure 2.

#### Treatment Phase

Participants in the aMCI group and the psMCI group completed a comparable number of minutes of BrainHQ exercises during the treatment phase. The groups also engaged in comparable numbers of exercises per cognitive domain during the treatment phase (ie, primarily the brain speed and attention domains), but the aMCI group engaged in twice as many different exercise types. In the aMCI group, most of the completed exercises were in the domains of brain speed (59.7%) and attention (35.4%), with some engagement in the memory (4.8%) and navigation (0.1%) domains. Ten different exercises were completed. In the psMCI group, all completed exercises were in the domains of brain speed (45.6%) and attention (54.4%) and included 5 exercises. Specific exercises that comprised >5% of the exercises by group are reported in Table 3.

**Table 3.** BrainHQ exercises by domain^a^.

BrainHQ domain	aMCI^b^ group BrainHQ exercises	psMCI^c^ group BrainHQ exercises
Treatment phase		
Brain Speed	Eye for Detail: 20.2% Hawk Eye: 15% Visual Sweeps: 13.1% Sound Sweeps: 11.4%	Eye for Detail: 18.7% Hawk Eye: 18.4% Visual Sweeps: 17.2%
Attention	Target Tracker: 19.2% Double Decision: 15.7%	Double Decision: 24.5% Target Tracker: 21.1%
			Follow-up phase		
Brain Speed	Eye for Detail: 17.6% Hawk Eye: 8.3% Sound Sweeps: 6.6% Visual Sweeps: 6.6%	Eye for Detail: 12.9% Sound Sweeps: 10.2% Visual Sweeps: 7.9% Fine Tuning: 6.7%
Attention	Target Tracker: 13.2% Double Decision: 10.4% Divided Attention: 8.8%	Target Tracker: 9.5% Double Decision: 8.7%
Memory	—^d^	Memory Grid: 7.7% Syllable Stacks: 7.4%
Intelligence	—	Mind Bender: 7.5% Juggle Factor: 6.2%

a Exercises that comprised ≤5 of the exercises are not included.

b aMCI: amnestic mild cognitive impairment.

c psMCI: post-stroke cognitive impairment.

d Not applicable.

#### Follow-Up Phase

Twenty (95%) of 21 participants in the aMCI group completed BrainHQ exercises in the follow-up phase. In contrast, in the psMCI group, only 9 (64%) of 14 participants completed any BrainHQ exercise in the follow-up phase. The proportion of those who engaged in any BrainHQ exercise significantly differed for weeks 3 and 4 (Fisher exact *P*<.03) as well as across all weeks combined (Fisher exact *P*=.03). Nonparametric longitudinal analyses showed that the number of minutes of engagement with BrainHQ exercises did not significantly differ between the 2 groups (ATS=3.06; *P*=.08). However, these analyses also showed that the number of minutes that aMCI participants completed increased over time compared to that of the psMCI group (ATS=6.72; *P*=.001), which decreased over the follow-up period. See Table 2 for the median number of minutes per week.

The variety of exercise types completed during follow-up was similar to the treatment phase. In the aMCI group, most exercises were in the brain speed (39.7%) and attention (36.9%) domains, followed by memory (8.5%), intelligence (5.8%), people skills (5.4%), and navigation (3.7%). A total of 28 different exercises were completed during the follow-up phase (Table 3). In the psMCI group, 38.2% of exercises during follow-up were in the domain of brain speed, followed by attention (24.5%), memory (19.7%), intelligence (13.7%), and people skills (3.9%). Fifteen different exercises were completed during the follow-up phase.

The aMCI group completed 2915 total exercises in the follow-up period. Of those, 62.5% were completed using the personal trainer option, and 48.5% were completed via the *a la carte* option in which participants chose the exercise, with 11 (55%) of 20 participants using the *à la carte* option for at least some follow-up-phase exercises. In comparison, of the 1224 exercises in the follow-up period that the psMCI group completed, only 1.7% of the exercises were completed via the *à la carte* option; only 1 (11%) of 9 psMCI participants who completed BrainHQ exercises during follow-up used the *à la carte option*.

In terms of device used, in the aMCI group, most exercises (60.5%) were completed on an iPad, followed by 22.6% completed via a web browser, 11.8% completed on an iPhone, and 5.1% completed on an Android device. Similarly, in the psMCI group, most exercises were completed using an iPad (41.3%), followed by 33.7% completed via a web browser, 24.2% via an Android device, and 0.8% via an iPhone.

### BrainHQ Acceptability

Medians and interquartile ranges for the items from the study-specific experience questionnaires are reported in Table 4 for the aMCI group and Table 5 for the psMCI group. Of note, although not all participants engaged with BrainHQ during the follow-up phase, all participants provided ratings of their experience immediately posttreatment. Thus, in Table 4, we present ratings separately for all participants post-treatment, all participants post-study, and the subset of participants who engaged in any BrainHQ during follow-up.

**Table 4.** aMCI^a^ group (n=21) BrainHQ experience questionnaire data.

Question	Post-TMS^b^ (n=21), median (IQR)	Post-study (n=21), median (IQR)	Post-study BrainHQ Engagers (n=20), median (IQR)
Rating scale (1=made things a lot worse, 2=made things somewhat worse, 3=made no difference, 4=made things somewhat better, 5=made things a lot better)			
How much did this treatment help with your mood?	4 (3-5)	4 (3-5)	4 (3-4)
How much did this treatment help with your cognitive functioning?	4 (3-5)	4 (3-4)	4 (3-5)
Rating scale (1=strongly disagree, 2=disagree, 3=neutral, 4=agree, 5=strongly agree)			
I became tired of doing BrainHQ exercises	2 (1-3)	2 (1-3)	2 (1-3)
I got frustrated using BrainHQ	2 (1-3)	3 (1-4)	3 (1-4)
I feel the BrainHQ exercises were appropriately challenging	5 (4-5)	5 (4-5)	5 (4-5)
I tried my best on all the BrainHQ exercises	5 (5-5)	5 (5-5)	5 (4.25-5)
I thought the BrainHQ exercise were fun	4 (4-5)	4 (3-4)	4 (3-4)
Rating scale (1=strongly disagree, 2=disagree, 3=neutral, 4=agree, 5=strongly agree)			
The research coordinators followed up with me to make sure I was completing my BrainHQ exercises	5 (5-5)	5 (4-5)	5 (4-5)
I understood what to do for each BrainHQ exercise	4 (4-5)	4 (4-5)	4 (3-5)
The research coordinators adequately explained how to use BrainHQ	5 (5-5)	5 (4-5)	5 (4-5)
I feel the BrainHQ website was easy to navigate	4 (4-5)	4 (4-5)	4 (4-5)
I was able to access the BrainHQ exercises at home without any problems	—^c^	5 (4-5)	5 (4-5)
I was satisfied with the feedback I got from BrainHQ about my performance on the exercises	5 (4-5)	4 (4-5)	4 (4-5)

a aMCI: amnestic mild cognitive impairment.

b TMS: transcranial magnetic stimulation.

c Not applicable.

**Table 5.** psMCI^a^ group (n=14) BrainHQ experience questionnaire data.

Question	Post-TMS^b^ (n=14), median (IQR)	Post-study (n=14), median (IQR)	Post-study BrainHQ engagers (n=9), median (IQR)
Rating scale (1=not at all, 2, 3=somewhat, 4, 5=very much so)			
The BrainHQ exercises helped improve my day-to-day activity levels and overall functioning.	3 (2-4)	3 (3-5)	3 (3-5)
The BrainHQ exercises helped improve mental abilities (eg, concentration, remembering, thinking, or making decisions).	3.5 (3-5)	4 (3-5)^c^	4 (3-4.75)^d^
Rating scale (1=not at all, 2, 3=somewhat, 4, 5=very much so)			
The BrainHQ sessions were overly time consuming and interfered with my daily life/responsibilities.	1 (1-1)	1 (1-1)	1 (1-1.5)
The BrainHQ sessions were overall difficult and burdensome.	1 (1-2)	2 (1-2)	2 (1-3)
I became bored with the BrainHQ training exercises.	1 (1-1)	1 (1-2)	1 (1-2.5)
I was engaged and tried my best to do well on the BrainHQ training exercises.	5 (5-5)	5 (5-5)	5 (5-5)
I was motivated to engage with and complete the BrainHQ training exercises.	5 (5-5)	5 (5-5)^c^	5 (4-5)
I enjoyed the BrainHQ training exercises.	5 (4-5)	4.5 (4-5)	4 (4-5)
Rating scale (1=not at all, 2, 3=somewhat, 4, 5=very much so)			
I would participate in studies that involve completing tasks similar to the BrainHQ training exercises.	5 (4.25-5)	5 (4.25-5)^c^	5 (4.5-5)
I am interested to learn what the researchers discover about the effects of the BrainHQ exercises on problems similar to mine.	5 (5-5)	5 (5-5)^c^	5 (5-5)
I am interested to learn more about the BrainHQ training exercises.	5 (5-5)	5 (4-5)	5 (4.5-5)
I would be interested in continuing BrainHQ exercises.	5 (4-5)	5 (4-5)	5 (4.5-5)
It would be beneficial if the BrainHQ training exercises were offered to me by my regular treatment providers.	4 (3-5)	4 (3-5)	5 (3-5)
I would recommend the BrainHQ exercises to a friend who experiences similar problems.	5 (5-5)	5 (4.25-5)	5 (4.5-5)
The instructions for using the BrainHQ website were explained clearly and were easy to understand.	5 (4-5)	5 (5-5)^c^	5 (4.5-5)

a psMCI: post-stroke mild cognitive impairment.

b TMS: transcranial magnetic stimulation.

c n=13.

d n=8.

Participants in the aMCI group, on average, rated the BrainHQ exercises as making their cognitive functioning and mood “somewhat better” at both post-treatment and 4-week follow-up. The aMCI participants generally rated their experience with BrainHQ positively at both time points; they found the exercises fun, appropriately challenging, not tiresome or frustrating, and they indicated that they tried their best. Finally, aMCI participants rated their experience with the BrainHQ platform as positive, finding it easy to navigate and access and adequately explained, understanding what to do, and being generally satisfied with the feedback from the BrainHQ platform at both time points.

Participants in the psMCI group generally rated the BrainHQ exercises as “*somewhat*” to “*very much so*” helpful in improving mental abilities and everyday functioning both post-treatment and at 4-week follow-up. psMCI participants also rated their experience with BrainHQ positively in terms of their motivation, trying their best, and enjoyment, and overall did not find BrainHQ exercises time-consuming, difficult or burdensome, or boring at both time points. On average, psMCI participants rated the platform positively, finding that it was easy to understand and that they had interest in continuing, interest in learning more about it or participating in similar studies, and that they would recommend the platform. Ratings of BrainHQ were similar posttreatment and post 4-week follow-up, despite somewhat limited engagement with BrainHQ in the follow-up period.

## Discussion

### Principal Findings

These 2 pilot studies provide preliminary support for the feasibility and acceptability of interleaving CCT with accelerated iTBS for people with MCI, given that there was comparably high engagement in the BrainHQ exercises during the in-laboratory treatment phase across both groups. This was consistent with our hypothesis, given the structured nature of administration after each accelerated iTBS session. Although, on average, participants completed only approximately half of the possible 210 training minutes, this finding is somewhat expected. The 10‐ to 15-minute inter-session intervals used for delivering CCT also had to include time spent navigating the platform, transitioning in and out of BrainHQ from iTBS sessions, and taking breaks (eg, resting in between sessions and using the restroom). Future studies using intermittent CCT will need to account for these timing factors when planning training time.

The groups differentially engaged with BrainHQ during the 4-week unsupervised follow-up phase. The engagement rate increased in the aMCI group over the 4 weeks, with most participants independently engaging with BrainHQ in the follow-up phase. Engagement in the psMCI group was numerically lower, although not statistically significant, and decreased over time, with the majority of participants not engaging with BrainHQ in follow-up weeks 3 and 4. Generally, this indicates that ecological adherence to CCT following iTBS may be feasible for many persons with MCI, although some patients may require more support (eg, regular reminders, in clinic, with care partner) to reach CCT engagement goals following iTBS. Notably, while several in the aMCI group and some in the psMCI group met the prescribed number of minutes in the follow-up phase, no participant in either group completed daily BrainHQ practice (ie, all 28 d). This may suggest that a minute-based target may be more attainable than a day-based one or that daily training may not be feasible in these populations. For both groups, the most commonly completed exercises were in the domains of attention and brain speed in both the treatment and follow-up phases. During the follow-up phase, the psMCI group engaged in somewhat more memory and intelligence exercises than those with aMCI; however, differences in exercise choice are difficult to interpret, given the lower engagement in this group. Finally, despite this differential engagement, both groups rated the acceptability of the BrainHQ exercises positively.

One of the most salient findings was that during the self-administered follow-up phase, the aMCI group, on average, completed closer to the recommended BrainHQ engagement time of 140 minutes per week, whereas the psMCI group, on average, did not complete the prescribed number of minutes, and engagement decreased over time. Likely contributors to this observed difference in ecological adherence are differences in study design. Notably, the aMCI study provided weekly email reminders to complete the daily CCT, whereas the psMCI study did not. These findings suggest that in cognitively impaired samples, the use of regular reminders and other remote supports may be necessary to support completion of CCT outside of the laboratory setting. Another potential factor is that the aMCI group was offered the à la carte option during follow-up, whereas the psMCI group was instructed to continue using the personal trainer. Allowing aMCI participants to self-select exercises (via à la carte) may have invoked greater autonomy over their training experience, leading to increased adherence.

It is also possible that disease-specific factors could have contributed to discrepant engagement in CCT during the self-administered follow-up phase. The aMCI group may have had greater motivation, perceived benefit, or expectancy of benefit from CCT, given that these participants knew that, given the neurodegenerative nature of AD, they would likely progressively decline. In comparison, the psMCI group, while still experiencing objective impairments, would have already experienced significant spontaneous recovery from their initial deficits due to the stroke, with a prognosis of stability of functioning if other vascular events are avoided. The type of cognitive deficits experienced by the 2 groups may have also contributed. As expected, the aMCI group had primarily amnestic impairment along with set-shifting and semantic fluency difficulties. In comparison, the psMCI group performed significantly worse on a test that involves cognitive processing speed and visuo-motor organization (ie, coding), which are processes required for completion of all BrainHQ exercises. The psMCI group also had more variable deficits, which is expected given that cognitive sequelae poststroke are heterogeneous. Additionally, while significant deficits that would affect the ability to complete cognitive tasks were exclusionary, mild visual and motor deficits are not uncommon post-stroke [35]. Most BrainHQ exercises, and particularly those in the domains of attention and processing speed, which were most often completed in this study, are visual in nature and require a fast response with the mouse or a finger. Therefore, the psMCI compared to the aMCI group may have found these exercises more challenging as a function of sensorimotor impairments that compounded the cognitive demands of the exercises, thus impacting their engagement.

This is the first study to our knowledge to elicit feedback on participants’ experience with the BrainHQ platform interleaved with accelerated iTBS. Although the study-specific surveys differed, limiting direct comparisons, results from both surveys generally indicated that participants in both groups found it relevant and potentially beneficial to their functioning, enjoyable and not excessively burdensome, and feasible for use during both treatment and post-treatment periods. The psMCI group rated BrainHQ favorably despite limited engagement in the follow-up post-treatment phase, so this questionnaire did not aid in identifying why engagement was lower compared to that in the aMCI group. Future research using qualitative and mixed methods may elucidate the factors driving differential engagement, which could inform CCT delivery in these populations. Despite the small-scale nature of these pilot studies, our findings highlight the importance of individualizing interventions to the specific population and underscore the need to avoid treating all cases of MCI as homogeneous syndromes when planning treatment approaches.

### Comparisons to Prior Work

Prior work investigating combined neuromodulation (eg, rTMS or transcranial direct current stimulation) and cognitive training has provided some additional insights into these combined interventions in the context of AD [36-38] and poststroke [39]. Although a growing literature supports the potential additive benefit of combining noninvasive brain stimulation (including rTMS) with cognitive training (including CCT; [40,41]), notably missing from the literature are studies refining methodologies for such combined interventions. Systematic reviews of the CCT literature demonstrate that CCT methodology is quite heterogeneous in terms of platform, training exercises, dose, and setting of administration [12,42]. While there is some evidence of the benefit of CCT to the same cognitive construct, there is limited support of benefits to different cognitive constructs and everyday activities [16]. However, many of these studies may not have been dosed for efficacy and used platforms with limited empirical support [21]. CCT was not explicitly dosed for efficacy in the current feasibility studies; however, a “gold standard” dose to elicit change has not been established [21]. Overall, the mixed literature and varied methodology suggest that there needs to be more research to establish which CCT methodology is most efficacious as well as how to deploy it in a way to elicit continued engagement (eg, increasing motivation or incentives and improved gamification). Moreover, more work is needed to identify the optimal method for combined delivery of CCT with other interventions, such as rTMS, particularly given that some methods (eg, required dose and most beneficial timing) may differ from CCT alone.

### Limitations

The studies presented here have some limitations and reveal several opportunities for future work. First, given that these were pilot studies focused on the safety, feasibility, and acceptability of combined accelerated iTBS and CCT, the intervention components were not explicitly dosed for efficacy. However, as higher doses (ie, increased treatment demands or durations) may impact participants’ engagement, continued consideration of feasibility and acceptability in future efficacy trials is necessary. Relatedly, the design of these studies did not include sham conditions, randomization, or blinding of participants or experimenters for either accelerated iTBS or CCT. Future randomized controlled trials that assess additive or synergistic effects of rTMS (accelerated iTBS or otherwise) and CCT are therefore needed. Second, both groups were predominantly non-Hispanic White with high levels of educational attainment. To ensure that research is representative of the general population, it is imperative that future research include participants from diverse sociodemographic backgrounds with varying levels of technology familiarity (which was not explicitly assessed in these studies). Third, people with aMCI and a history of stroke often experience neuropsychiatric symptoms, most commonly depression [43,44]; however, participants in our studies did not have clinically significant neuropsychiatric symptoms. Given that depressive symptoms are associated with poorer adherence to multidomain behavioral interventions (including cognitive training) in older adults [45], feasibility and acceptability of combined rTMS and CCT may be lower in people with MCI and comorbid neuropsychiatric symptoms. Alternatively, as rTMS to the l-dlPFC is US Food and Drug Administration–approved to treat depression, and both rTMS [46] and CCT [47] may improve depression or apathy in MCI, engagement in combined rTMS and CCT may instead be high in those with MCI and depression if they experience such benefits from the treatment. Therefore, our findings warrant replication in people with MCI and comorbid neuropsychiatric conditions.

### Conclusions

In conclusion, this study provides initial evidence that accelerated iTBS to the l-dlPFC interleaved with CCT during treatment is feasible and acceptable to many patients with MCI. However, more limited engagement in the follow-up phase suggests that the feasibility of self-directed CCT may vary across people with MCI and by CCT delivery format (eg, personal trainer vs. à *la carte),* and indicates that regular reminders may be beneficial. These findings suggest that personalized treatment approaches and methods to reinforce CCT may enhance feasibility and adherence. Our results can directly inform future trials aimed at optimizing treatments that combine neuromodulatory and CCT interventions. Subsequent randomized controlled trials that evaluate whether combined rTMS and CCT yields a synergistic or additive impact on MCI symptoms are warranted.

## Supplementary material

10.2196/81437 Multimedia Appendix 1 Summary of the Template for Intervention Description and Replication (TIDieR) checklist.

## Abbreviations:

AD: Alzheimer's disease
aMCI: amnestic mild cognitive impairment
ATS: ANOVA-type statistic
CCT: computerized cognitive training
iTBS: intermittent theta burst
l-dlPFC: left dorsolateral prefrontal cortex
MCI: mild cognitive impairment
MUSC: Medical University of South Carolina
NIA-AA: National Institute on Aging and Alzheimer's Association
NIH: National Institutes of Health
NINDS: National Institute of Neurological Disorders and Stroke
NMDA: N-methyl-d-aspartate
PROMIS: Patient-Reported Outcomes Measurement Information System
psMCI: post-stroke mild cognitive impairment
rTMS: repetitive transcranial magnetic stimulation
TIDieR: Template for Intervention Description and Replication
