# Supplementary material for: Feasibility of Interleaving Computerized Cognitive Training With Repetitive Transcranial Magnetic Stimulation: Pilot Studies in Mild Cognitive Impairment Due to Alzheimer’s Disease and Stroke
Source: JMIR Neurotechnol. 2026 Jul 15;5:e81437. doi: 10.2196/81437 (PMC13372265; doi:10.2196/81437)
Supplement: Multimedia Appendix 1 [file neuro-v5-e81437-s001.docx]

**Supplementary Table 1. Summary of the Template for Intervention Description and Replication (TIDieR) Checklist**

| **Checklist Item** | **Checklist Descriptor** | **Summary** |
| --- | --- | --- |
| 1 | Name of intervention | The BrainHQ (https://www.brainhq.com/) computerized cognitive training platform was the primary intervention investigated in this analysis which was completed in conjunction with accelerated intermittent theta burst (iTBS) repetitive transcranial magnetic stimulation (rTMS) to the left dorsolateral prefrontal cortex. |
| 2 | Rationale | It is hypothesized that cognitively stimulating computerized cognitive training (CCT), such as BrainHQ, can enhance performance on similar tasks via neuroplasticity. In addition to rTMS-induced neuroplasticity, it is plausible that rTMS in combination with CCT may yield more improvement than rTMS or CCT alone. Few studies have examined the feasibility or acceptability of combined CCT and rTMS interventions, particularly in individuals with mild cognitive impairment. |
| 3 | Materials | BrainHQ was completed on a study-owned iPad (10.2 inch iPad, 8^th^ generation, iOS 16.4) during the treatment days and then on the participants preferred device during follow up. |
| 4 | Procedures | BrainHQ-defined training categories include attention, brain speed, memory, people skills, intelligence, and navigation (specific exercises that participants completed are detailed in the results section). Training exercises become more difficult as participants improve on the exercises and participants are provided feedback on their performance on each activity and over time. During the treatment phase, participants used the “personal trainer” option that selects exercises for the participant based on past performance on exercises. During the follow-up phase, participants could use either the personal trainer or the “a la carte” option in which participants can select which exercise they would like to complete. |
| 5 & 6 | Who provided intervention and mode of delivery | BrainHQ was self-administered during the treatment and follow-up phases. During the treatment phase, staff were available in person and helped participant get set up on the device, answer questions, and encouraged use. |
| 7 | Location | During the treatment phase, BrainHQ was completed in clinic. During the follow-up phase, BrainHQ was completed at the participants' preferred location. |
| 8 | When and how much | During the seven 10-15 minute breaks between each of iTBS sessions on the three treatment days, all participants were asked to complete BrainHQ exercises; participants were then asked to complete at least 20 minutes of BrainHQ exercises per day during the 4-week follow-up period. |
| 9 | Tailoring | BrainHQ uses adaptive algorithm to tailor training to the participants level and participants were able to self-tailor their training during the follow-up phase (i.e., use of "personal trainer" or the "a la carte" option) given their preference; there was no study investigator-initiated prescription outside of these options. |
| 10 | Modifications | None |
| 11 & 12 | Planned and actual fidelity | During treatment, staff was there to support BrainHQ adherence; during follow-up, staff called participants once a week during the 4-week follow-up to ask about and encourage BrainHQ usage. All participants completed at least some BrainHQ during the treatment phase; adherence to the recommended 140 minutes of BrainHQ exercises per week during the follow-up phase was variable. See results for details. |
